# Child hepatitis of unknown origin may be due to insufficient understanding of adenovirus pathogenicity

**DOI:** 10.1002/hep4.2020

**Published:** 2022-06-06

**Authors:** Yong Qi, Wenping Gong

**Affiliations:** ^1^ Huadong Research Institute for Medicine and Biotechniques Nanjing China; ^2^ Tuberculosis Prevention and Control Key Laboratory/Beijing Key Laboratory of New Techniques of Tuberculosis Diagnosis and Treatment, Senior Department of Tuberculosis The Eighth Medical Center of PLA General Hospital Beijing China

## Abstract

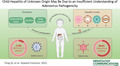

To the editor,

Child hepatitis of unknown origin has drawn attention worldwide. As of May 12, 2022, 492 cases of acute hepatitis, including 12 deaths, have been reported in children aged 16 years and younger.^[^ ^1^ ^]^ These cases showed initial symptoms of gastroenteritis illness followed by jaundice and acute hepatitis. Hepatitis viruses A–E have been ruled out. In addition, most patients did not have a fever and are less than 5 years of age.^[^ ^1^ ^]^ Nucleic acid testing revealed 59.6% (90/151 tested cases) were adenovirus (AdV) positive and 11.6% (20/173 polymerase chain reaction‐tested cases) were severe acute respiratory syndrome (SARS)‐coronavirus 2 (CoV‐2) positive, with 19 coinfection cases.

A recent correspondence hypothesized that the hepatitis in children could be a consequence of the coexistence of AdV and SARS‐CoV‐2, leading to excessive interferon‐γ‐mediated apoptosis of hepatocytes.^[^ ^2^ ^]^ The paper indicated superantigens of SARS‐CoV‐2 should be investigated, which in our opinion, may be misleading.

Based on the available evidence, AdV infection is undoubtedly the direct and main cause. AdV can cause gastrointestinal symptoms even when the primary involvement site is the respiratory tract, particularly in young children,^[^ ^3^ ^]^ and gastrointestinal affinity AdV‐40 and AdV‐41 infections show predominant symptoms of gastroenteritis or diarrhea and rare complications of hemorrhagic colitis, hepatitis, cholecystitis, or pancreatitis.^[^ ^3^ ^]^ This is totally consistent with the observed symptoms in these cases with a high AdV‐positive rate and also explains why other AdV types were identified. Moreover, due to lack of humoral immunity, children less than 4 years old occupy more than 80% of diagnosed AdV infections,^[^ ^3^ ^]^ which is consistent with the predominant ages of 3–5 in children with acute hepatitis.

We propose that severe AdV‐caused hepatitis in humans may have been circulating for a long time. It could be questioned why the disease was not monitored before. First, AdV hepatitis was typically neglected by physicians. A low incidence of severe hepatitis of unknown origin in young children existed before, but the causes lacked in‐depth investigations after ruling out hepatitis viruses A–E. Second, coinfection of SARS‐COV‐2 and AdV was detected in some cases, and this may have aggravated the severity of AdV‐caused hepatitis, as has occurred in hepatitis B virus‐caused hepatitis. The role of coronavirus disease 2019 (Covid‐19) here is simply to amplify the number of cases of severe hepatitis to be noticed. Third, during the pandemic, public health agencies increased their surveillance of infectious diseases, leading to the eventual discovery and worldwide attention of the disease. Rather, a case that first reported AdV hepatitis in an adult who was immunocompetent in the United States may extend the traditional view that AdV causes severe hepatitis only in patients who are immunocompromised.^[^ ^4^ ^]^ The classical innate immune cells, including natural killer cells, neutrophils, and Kupffer cells, may be responsible for the development of AdV hepatitis‐mediated acute liver toxicity through cytokine–chemokine crosstalk.^[^ ^5^ ^]^

More than 100 AdV types have been identified, with novel and recombinant types emerging; most of these types have had insufficient investigation, especially their pathogenesis in children. Nevertheless, we suggest retrospective studies of non‐hepatitis viruses A–E‐caused hepatitis are needed along with a focus on the pathogenesis of emerging AdV types.

## AUTHOR CONTRIBUTIONS

Conceptualization, data curation, draft review, and editing: Yong Qi and Wenping Gong; Formal analysis and writing original draft: Yong Qi; Funding acquisition, methodology, data analysis: Wenping Gong.

## FUNDING INFORMATION

Beijing Municipal Science and Technology Commission, Grant: 19L2065; Chinese PLA General Hospital, Grant: QNC19047.

## CONFLICT OF INTEREST

Nothing to report.
